# Fucoxanthin attenuates LPS-induced acute lung injury via inhibition of the TLR4/MyD88 signaling axis

**DOI:** 10.18632/aging.202309

**Published:** 2020-12-11

**Authors:** Xiaoling Li, Riming Huang, Kaifeng Liu, Mingyue Li, Hui Luo, Liao Cui, Lei Huang, Lianxiang Luo

**Affiliations:** 1 Experimental Animal Center, Guangdong Medical University, Zhanjiang 524023, Guangdong, China; 2 Guangdong Provincial Key Laboratory of Food Quality and Safety, College of Food Science, South China Agricultural University, Guangzhou 510642, China; 3 The First Clinical College, Guangdong Medical University, Zhanjiang 524023, Guangdong, China; 4 Department of Pathology and Laboratory Medicine, Perelman School of Medicine, University of Pennsylvania, Philadelphia, PA 19126, USA; 5 The Marine Biomedical Research Institute, Guangdong Medical University, Zhanjiang 524023, Guangdong, China; 6 Marine Medical Research Institute of Zhanjiang, Zhanjiang 524023, Guangdong, China; 7 Guangdong Key Laboratory for Research and Development of Natural Drugs, Guangdong Medical University, Zhanjiang 524023, Guangdong, China; 8 Department of Molecular, Cell and Cancer Biology, University of Massachusetts Medical School, Worcester, MA 01605, USA

**Keywords:** acute lung injury, fucoxanthin, LPS, TLR4, MyD88, NF-κB

## Abstract

Acute lung injury (ALI) is a critical clinical condition with a high mortality rate. It is believed that the inflammatory storm is a critical contributor to the occurrence of ALI. Fucoxanthin is a natural extract from marine seaweed with remarkable biological properties, including antioxidant, anti-tumor, and anti-obesity. However, the anti-inflammatory activity of Fucoxanthin has not been extensively studied. The current study aimed to elucidate the effects and the molecular mechanism of Fucoxanthin on lipopolysaccharide-induced acute lung injury. In this study, Fucoxanthin efficiently reduced the mRNA expression of pro-inflammatory factors, including IL-10, IL-6, iNOS, and Cox-2, and down-regulated the NF-κB signaling pathway in Raw264.7 macrophages. Furthermore, based on the network pharmacological analysis, our results showed that anti-inflammation signaling pathways were screened as fundamental action mechanisms of Fucoxanthin on ALI. Fucoxanthin also significantly ameliorated the inflammatory responses in LPS-induced ALI mice. Interestingly, our results revealed that Fucoxanthin prevented the expression of TLR4/MyD88 in Raw264.7 macrophages. We further validated Fucoxanthin binds to the TLR4 pocket using molecular docking simulations. Altogether, these results suggest that Fucoxanthin suppresses the TLR4/MyD88 signaling axis by targeting TLR4, which inhibits LPS-induced ALI, and fucoxanthin inhibition may provide a novel strategy for controlling the initiation and progression of ALI.

## INTRODUCTION

Acute lung injury (ALI) is a disease of pulmonary edema and respiratory failure caused by the injury of capillary endothelial cells and alveolar epithelial cells. It is a critical clinical disease with high mortality [1]. So far, there is no effective treatment for ALI [2]. Thus, identification of effective therapeutic drugs is urgently needed for the treatment of patients with ALI. It has been reported that abnormal inflammatory factors are essential factors in the occurrence and development of ALI, such as tumor necrosis factor-α (TNF-α), interleukin-6 (IL-6), and interleukin-1β (IL-1β) [3]. As such, removing the excessive production of those cytokines by anti-inflammatory agents, including corticosteroids, such as methylprednisolone [4], dexamethasone [5], prednisolone [6], represented a promising strategy to prevent and treat ALI [7]. However, most of these drugs have no beneficial effect on ALI patients because of their low efficacy and severe side effects. Therefore, the treatment of ALI urgently needs new anti-inflammatory drugs with better efficacy and safety.

Lipopolysaccharide (LPS) is the main cell wall component of Gram-negative bacteria, which can cause the disorder of immune and inflammatory response. At present, it is widely used to induce the ALI model [8]. LPS initiates a variety of molecular intracellular signaling events, including the activation of nuclear factor kappa B (NF-κB) [9]. Macrophages are essential cells in the inflammatory process. LPS-activated macrophages, as the second level of the inflammatory cascade, release inflammatory cytokines, such as TNF-α, IL-6, IL-1β, and cyclooxygenase (COX2) [10].

The uniqueness and diversity of the marine environment lead to the production of active substances with unique functions and structures [11, 12]. In recent years, marine compounds have become an essential source of drug development [13]. Fucoxanthin is a carotenoid and was first extracted from brown seaweed (i.e., *Undaria pinnatifida, Saccharina japonica, Sargassum fulvellum*), which contains functional groups. Fucoxanthin has been reported various biological activities, such as anti-obesity, neuroprotective effect, and anti-cancer properties [14–17]. Recently, the anti-inflammatory effect of Fucoxanthin has been expanded [18]. Fucoxanthin reduced alcohol-induced inflammatory responses by activating the Nrf2-mediated pathway [19]. Fucoxanthin preventive anti-inflammatory effect in a mouse model of Sepsis is associated with the regulation of NF-κB signaling [20]. Fucoxanthin down-regulates the DSS-induced expression of COX-2 and NF-κB in mice [21]. Fucoxanthin inhibited LPS-induced overexpression of pro-inflammatory cytokines (IL-1β, IL-6, iNOS, COX-2, and TNF-α) via the AMPK/NF-κB signaling pathway [16]. Nevertheless, whether the Fucoxanthin has protective effects on ALI and the potential mechanisms remains to be unclear.

In the current study, we assess the effects of Fucoxanthin on the LPS-induced ALI cell model and investigate the effect of Fucoxanthin on LPS-induced ALI mouse model, and the signal transduction mechanism of its anti-inflammatory effect was also discussed.

## RESULTS

### Effect of fucoxanthin on RAW264.7 cells viability

**Figure 1:**
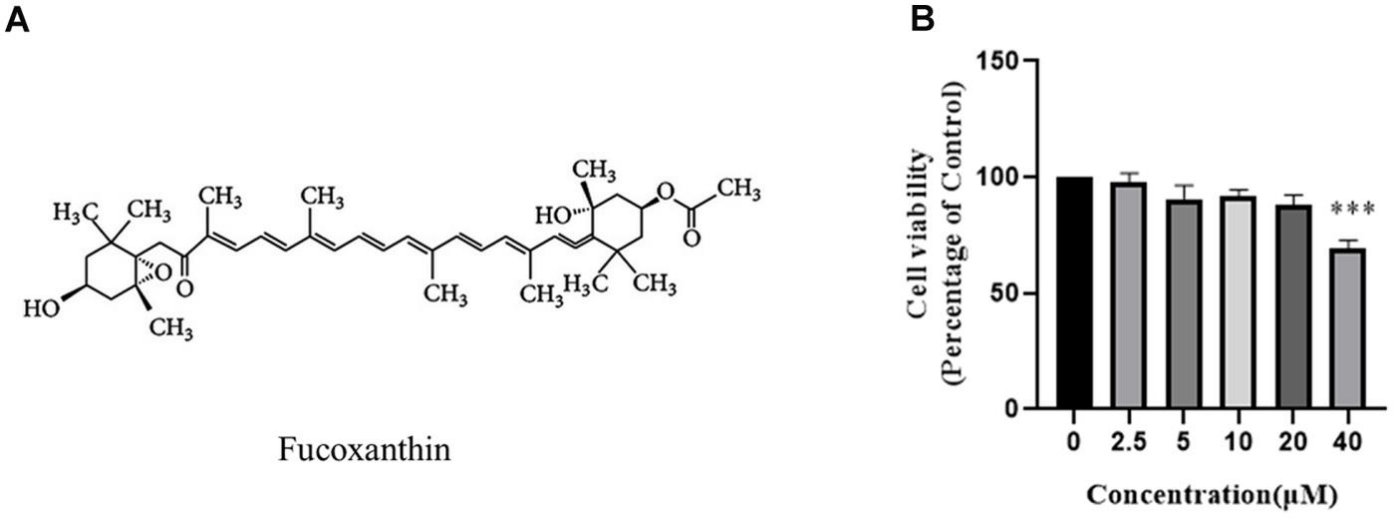
**Effect of Fucoxanthin on RAW264.7 cell viability.** (**A**) Chemical structure of Fucoxanthin. (**B**) The cells were stimulated with the indicated concentrations of Fucoxanthin (0,2.5,5,10,20,40uM) for 24h, and cell viability was determined using the MTT assay. ***P <0.001.

To assess the cytotoxic effect of Fucoxanthin on RAW264.7 cells, the cell viability was detected by CCK-8 assays, and the structure of Fucoxanthin was presented in Figure 1A. As shown in Figure 1B, RAW264.7 cells were treated with 0-20 μM Fucoxanthin for 24 hours had no cytotoxic effect, whereas cell viability was significantly reduced at the concentration of 40 μM.

### Fucoxanthin inhibits LPS-stimulated expression of COX-2 and iNOS in RAW264.7 cells

**Figure 2:**
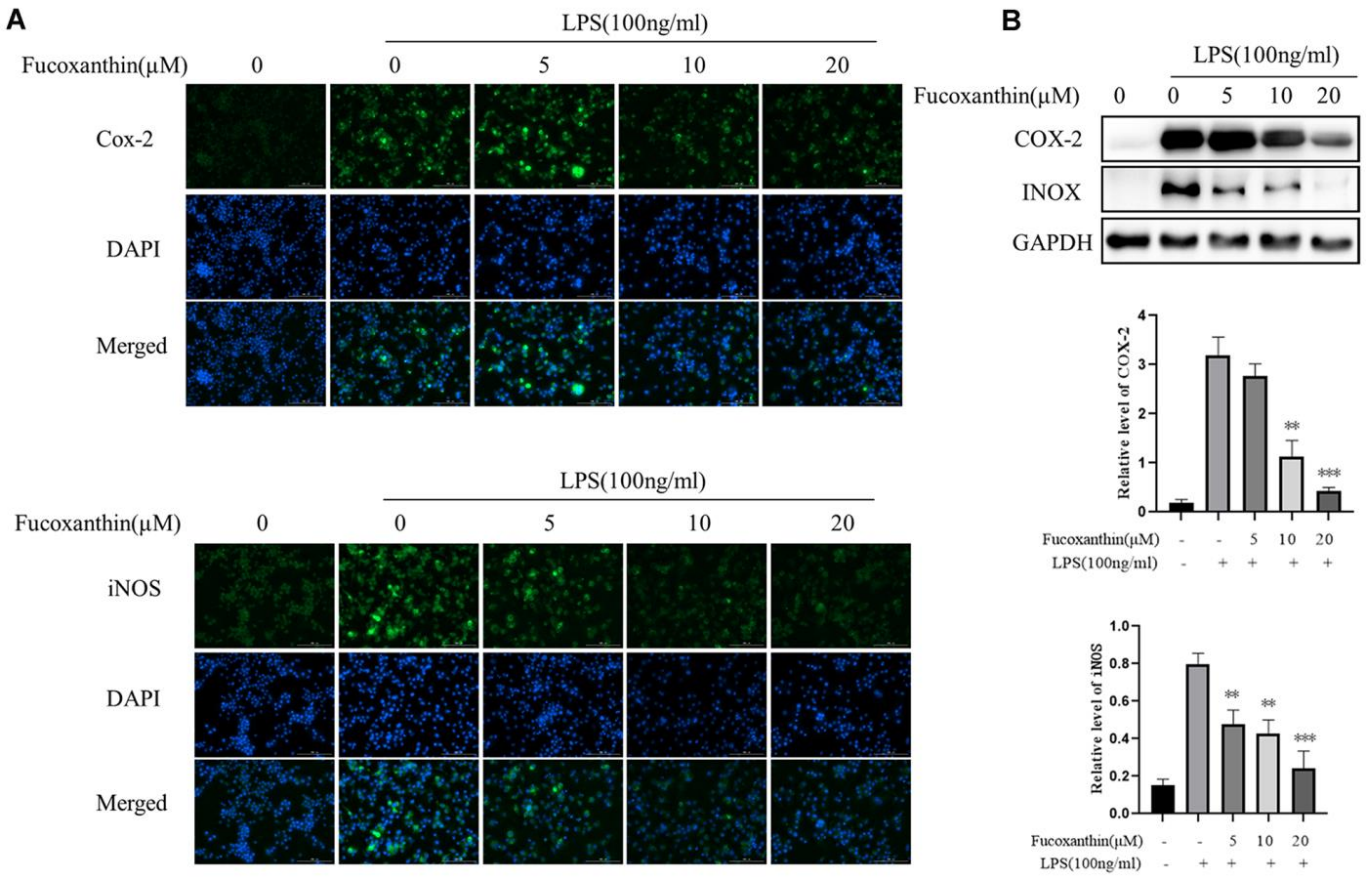
**Fucoxanthin down-regulated the expression of COX-2 and iNOS in LPS-activated RAW 264.7 macrophages.** (**A**) RAW264.7 cells were pretreated with the indicated concentrations of Fucoxanthin for 1 h before being stimulated with LPS for another 24 h. Cox-2 and iNOS were determined by immunofluorescence staining. DAPI-stained nuclei were indicated by blue fluorescence. Cox-2 and iNOS were indicated by green fluorescence. Scale Bar = 100 μm. (**B**) Cells were pretreated with the indicated concentrations of Fucoxanthin for 1 h before being stimulated with LPS (100ng/mL) for another 24 h. The expression levels of COX-2 and iNOS were determined by immunoblotting. *P < 0.05, **P < 0.01 compared with the LPS group.

iNOS is a pivotal downstream mediator of inflammation in various cell types [22]. Moreover, COX-2 is a critical inflammatory mediator [23]. Studies have shown that the expression of iNOS and cox-2 is significantly increased in LPS-induced macrophages [24]. We determined whether Fucoxanthin could modulate LPS-induced expression of COX-2 and iNOS in macrophages. As shown in Figure 2A, Fucoxanthin distinctly suppressed expression of COX-2 and iNOS in RAW264.7 cells induced by LPS with immunofluorescence. Furthermore, Western blot analysis also showed that Fucoxanthin could dramatically reduce the protein expression of COX-2 and iNOS induced by LPS (Figure 2B), indicating that Fucoxanthin is a potential drug against inflammation *in vitro*.

### Fucoxanthin inhibits LPS-stimulated expression of pro-inflammatory cytokines in RAW264.7 cells

**Figure 3:**
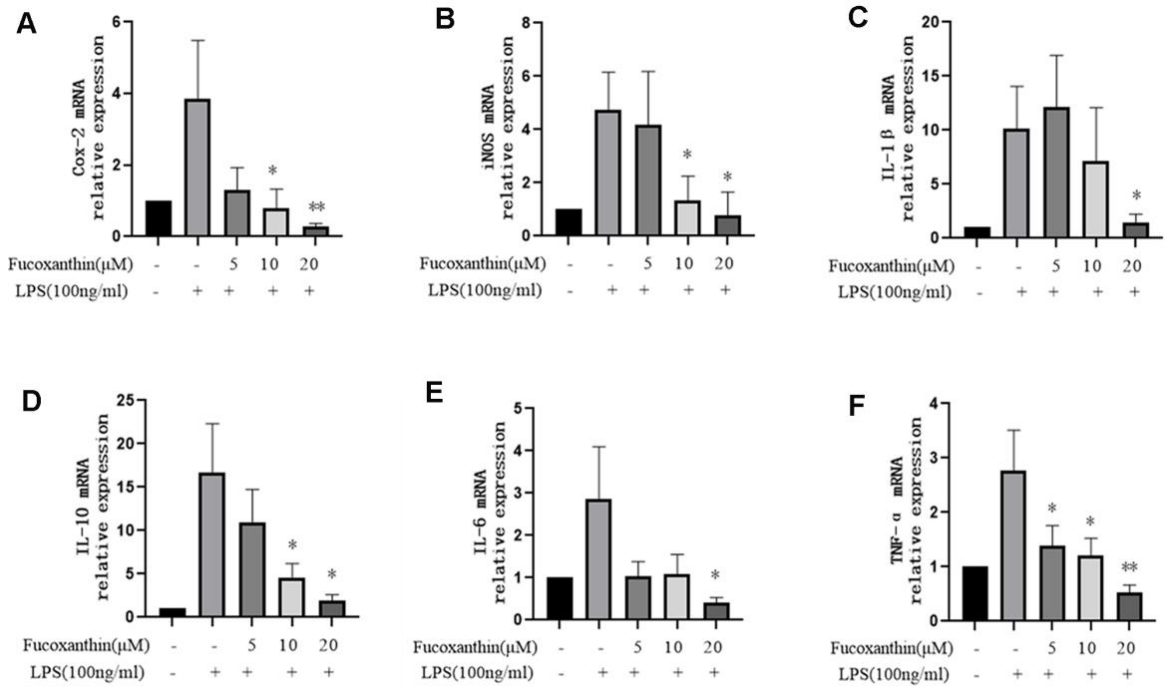
**Effects of Fucoxanthin on the mRNA level of pro-inflammatory cytokines in LPS-induced RAW264.7 cells.** The cells were pretreated with Fucoxanthin (5, 10, and 20 μM) for 1 h and then stimulated with LPS (100ng/mL) for 24 h. Untreated cells served as control. The total RNA was prepared, and the mRNA expression levels of (**A**) Cox-2, (**B**) iNOS, (**C**) IL-1β, (**D**) il-10, (**E**) IL-6, and (**F**) TNF-α were determined by qRT-PCR. The values presented are means ± SD. *P < 0.05, ^**^P < 0.01 versus LPS.

In order to further evaluate the anti-inflammatory activity of Fucoxanthin on LPS-stimulated macrophages, we examined its effect on the expression of pro-inflammatory cytokines stimulated by LPS, such as COX-2, iNOS, IL-10, IL-6, TNF-α, and IL-1β. We tested mRNA expression levels of these cytokines using the qRT-PCR method. Our results revealed that LPS dramatically increased the mRNA expression levels of COX-2, iNOS, IL-10, IL-6, TNF-α, and IL-1β in RAW264.7 cells compared with those in the control group, while pre-treatment with Fucoxanthin completely diminished the stimulatory effects of LPS in a dose-dependent manner (Figure 3A–3F).

### Fucoxanthin inhibits LPS-induced activation of NF-κB signaling in RAW264.7 cells

**Figure 4:**
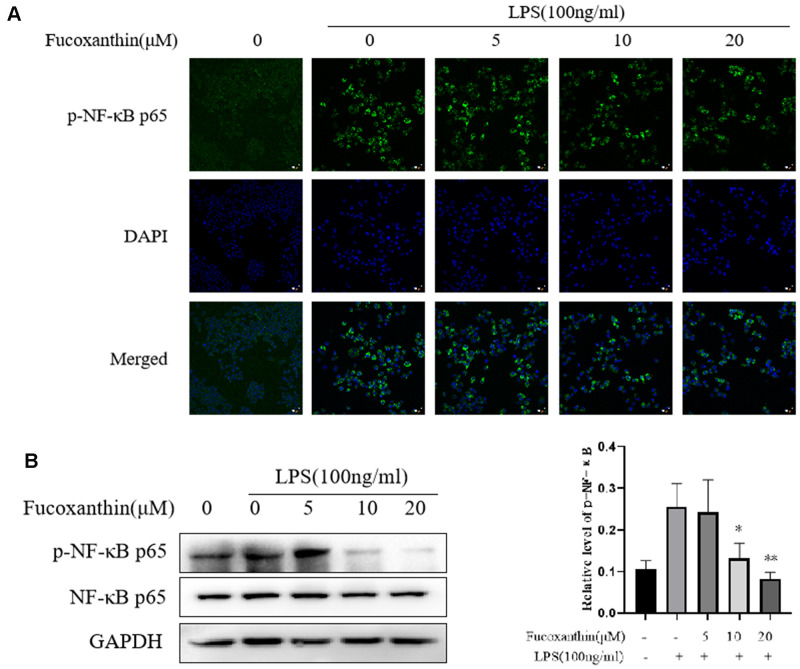
**Fucoxanthin suppresses LPS-induced activation of NF-κB signaling in RAW 264.7 cells.** (**A**) RAW264.7 cells were pretreated with the indicated concentrations of Fucoxanthin for 1 h before being stimulated with LPS for another 24 h. p-NF-κB was determined by immunofluorescence staining. DAPI-stained nuclei are indicated by blue fluorescence. NF-κB was indicated by red fluorescence, scale bar=200 μm. (**B**) The expression level of p-NF-κB p65 and NF-κB p65 was determined by immunoblotting. *P < 0.05, **P < 0.01 compared with the LPS alone group.

NF-κB is a crucial regulator and participated in the inflammatory process [25, 26]. Our results showed that Fucoxanthin significantly prevented activation of TLR4/MyD88 induced by LPS. Nevertheless, the activation of TLR4/MyD88 can directly affect the signal axis of NF-κB. As shown in Figure 4A, Fucoxanthin effectively blocked the nuclear translocation of NF-κB. To further confirm our results, we detected the effect of Fucoxanthin on the expression level of NF-κB by Western blotting. The results showed that Fucoxanthin only affected the expression level of p-NF-κB (p-p65), but not the expression level of total NF-κB (p65) (Figure 4B). These results indicate that Fucoxanthin prevents LPS-induced inflammatory responses by attenuating the release of pro-inflammatory cytokines through the inhibition of TLR4/MyD88/NF-κB activation.

### Potential anti-inflammatory mechanisms of fucoxanthin by network pharmacological analysis

**Figure 5:**
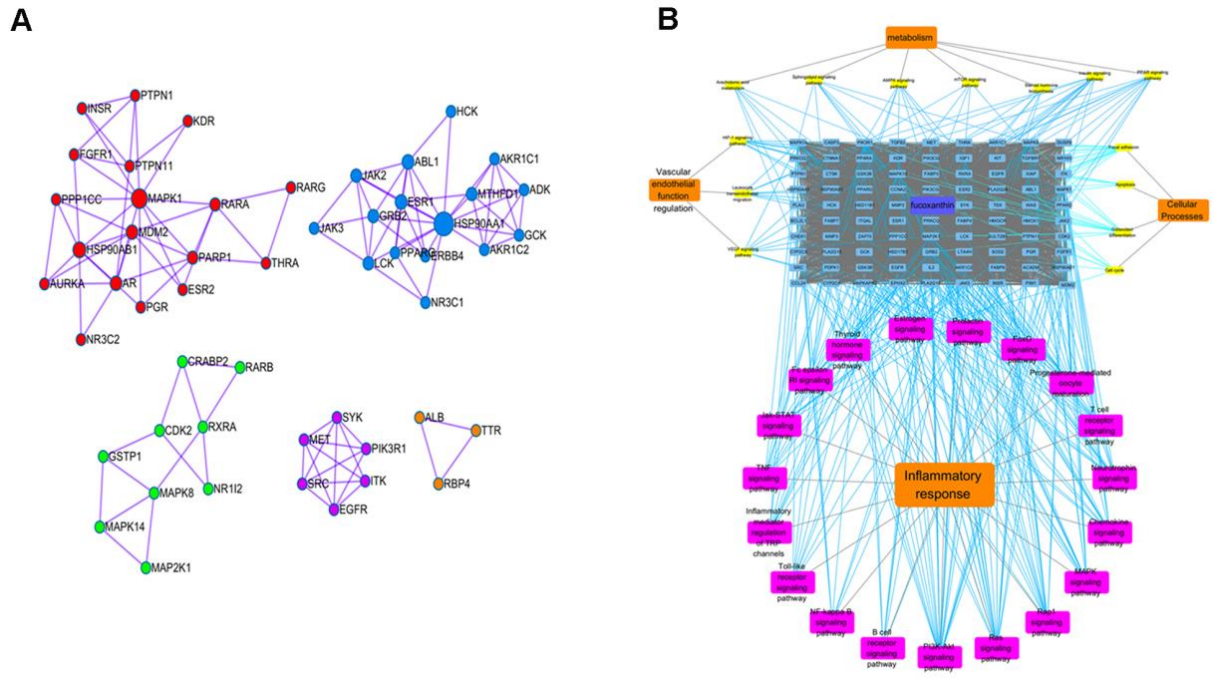
**Potential anti-inflammatory mechanisms of Fucoxanthin by network pharmacological analysis.** (**A**) Cluster analysis in MCODE. Different colors represent different clusters. (**B**) Target-pathway interaction diagram. The blue squares above represent fucoxanthin anti-ALI target, yellow squares represent vascular endothelial function regulation, metabolism, and cellular processes-related pathways, and the purple squares represent inflammatory response-related pathways.

The targets of Fucoxanthin and ALI were screened by Z value and corrected by Uniprot. Eighty-four human protein gene symbols of Fucoxanthin and 5442 human protein gene symbols of ALI were obtained. One hundred sixty-three targets of Fucoxanthin against ALI proteins were obtained after the intersection. The MCODE plug-in was used to cluster, and the cluster was divided into 5 clusters, which meant that metabolic pathways were interlaced and regulated mutually. MAPK8 is the core target in cluster 1. HSP90AA1 is the core target in cluster2. PIK3R1, SRC, and EGFR are classified in the same cluster (Figure 5A). Through KEGG pathway enrichment analysis of common-target and PPI networks, 33 significant signaling pathways (P-value ≤ 0.05) were screened as fundamental action mechanisms of Fucoxanthin on ALI. The signal pathways involved anti-inflammation, vascular endothelial function regulation, metabolic regulation, and cellular processes regulation. Among them, there are 3 related to vascular endothelial function regulation, 7 related to metabolism, 19 related to inflammatory response, and 4 related to cellular processes (Figure 5B). The molecular signal pathway of Fucoxanthin against inflammation was closely related to inflammation-based pathways, such as the TNF signaling pathway, PI3K-Akt signaling pathway, and Toll-like receptor signaling pathway. This evidence uncovers that the anti-inflammatory effect is the most robust in the Target-Function map, and it is the primary function of the anti-ALI related targets of Fucoxanthin. It is proved that the anti-inflammatory effect of Fucoxanthin is the strongest in the target function diagram, and is also the primary function of Fucoxanthin in anti-ALI related targets.

### Fucoxanthin directly binds to TLR4 and prevents the TLR4/MyD88/NF-κB signaling pathway

**Figure 6:**
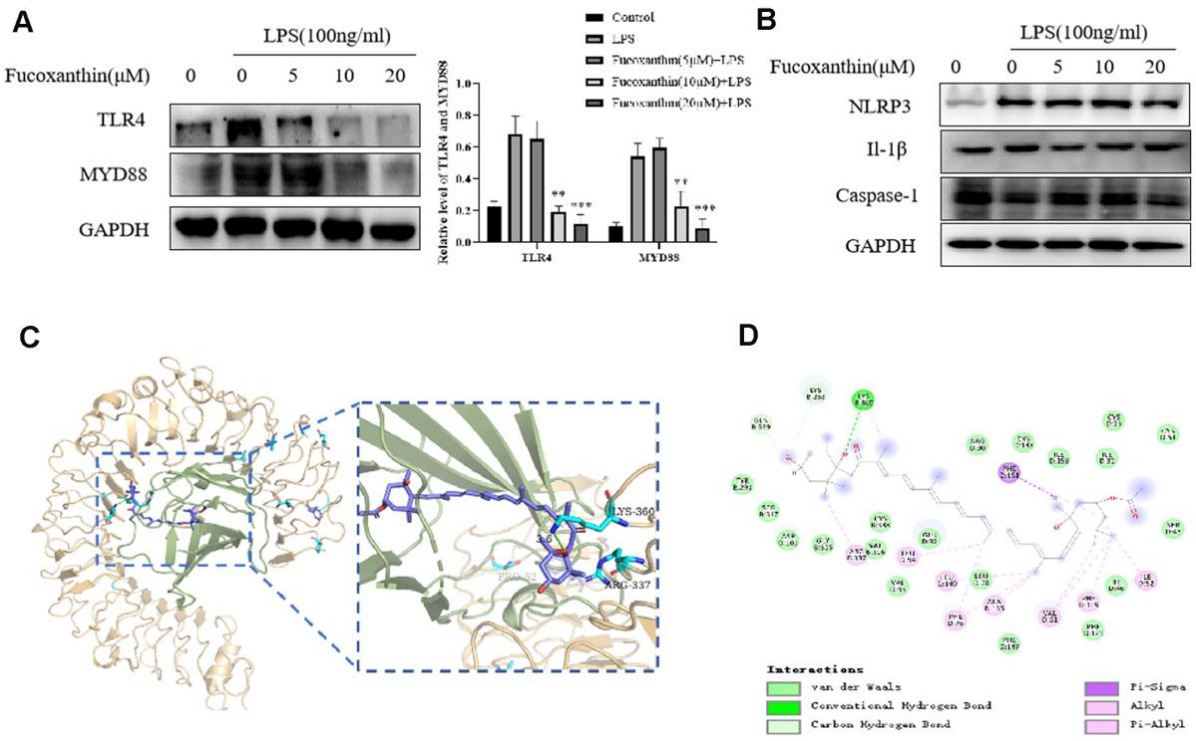
**Fucoxanthin directly binds TLR4 inhibiting the TLR4/MyD88/NF-κB signaling pathway.** (**A**) Western blotting analysis of TLR4 and MYD88 for RAW 264.7 cells pretreated with different concentrations of the Fucoxanthin for 1h followed by LPS treatment (100ng/ml) for another 24 h. (**B**) The expression level of NLRP3, Il-1β, and caspase-1 were determined by immunoblotting (**C**) Schematic representations 3D of the binding interactions between the mouse TLR4/MD-2 complex active site and Fucoxanthin. MD-2 was shown in a light green ribbon and TLR4 in a light orange ribbon (left) close-up view of the predicted interaction between Fucoxanthin and the MD-2-binding site TLR4-MD-2 complex. MD-2 was shown in light green and TLR4 in light orange; moreover, the hydrogen bond is shown in green. (**D**) Schematic representations 2D of the binding interactions between the mouse TLR4/MD-2 complex active site and Fucoxanthin.

Upon the stimulation of LPS, TLR4 initiates series of inflammatory responses [27]. Here, we try to determine further whether Fucoxanthin influences the TLR4/MyD88 signaling pathway. As we expected, Fucoxanthin effectively suppressed the TLR4/MyD88 signaling pathway (Figure 6A). In addition, previous studies have shown that inflammatory body-induced cytokines play a vital role in the occurrence and development of ALI, and inhibition of the NLRP3 signal pathway can reduce LPS-induced ALI [28, 29]. We tried to detect the effects of Fucoxanthin on other inflammatory signaling pathways. The results showed that Fucoxanthin did not affect the NLRP3 signal pathway (Figure 6B). Based on the evidence, we speculated that a potential binding mode for Fucoxanthin is that it might disrupt the TLR4 signaling by interacting with MD-2, complex as TLR4 [30]. A molecule docking search was carried out to determine whether there is a required binding mode at the interface between Fucoxanthin and TLR4/MD-2 protein. The result shows that Fucoxanthin may well bind to the TLR4/MD-2 complex and can form interaction with LYS-360, PHE-151, ARG-337, and PHE-119 (Figure 6C, 6D). Fucoxanthin can bind to an accessory protein MD-2 of the mouse TLR4/MD-2 complex via hydrogen bonding and hydrophobic interactions. These results suggested that Fucoxanthin may be the interference factor of TLR4/MD-2 protein-protein interaction.

### Fucoxanthin attenuates the inflammation in LPS-treated ALI mouse model

**Figure 7:**
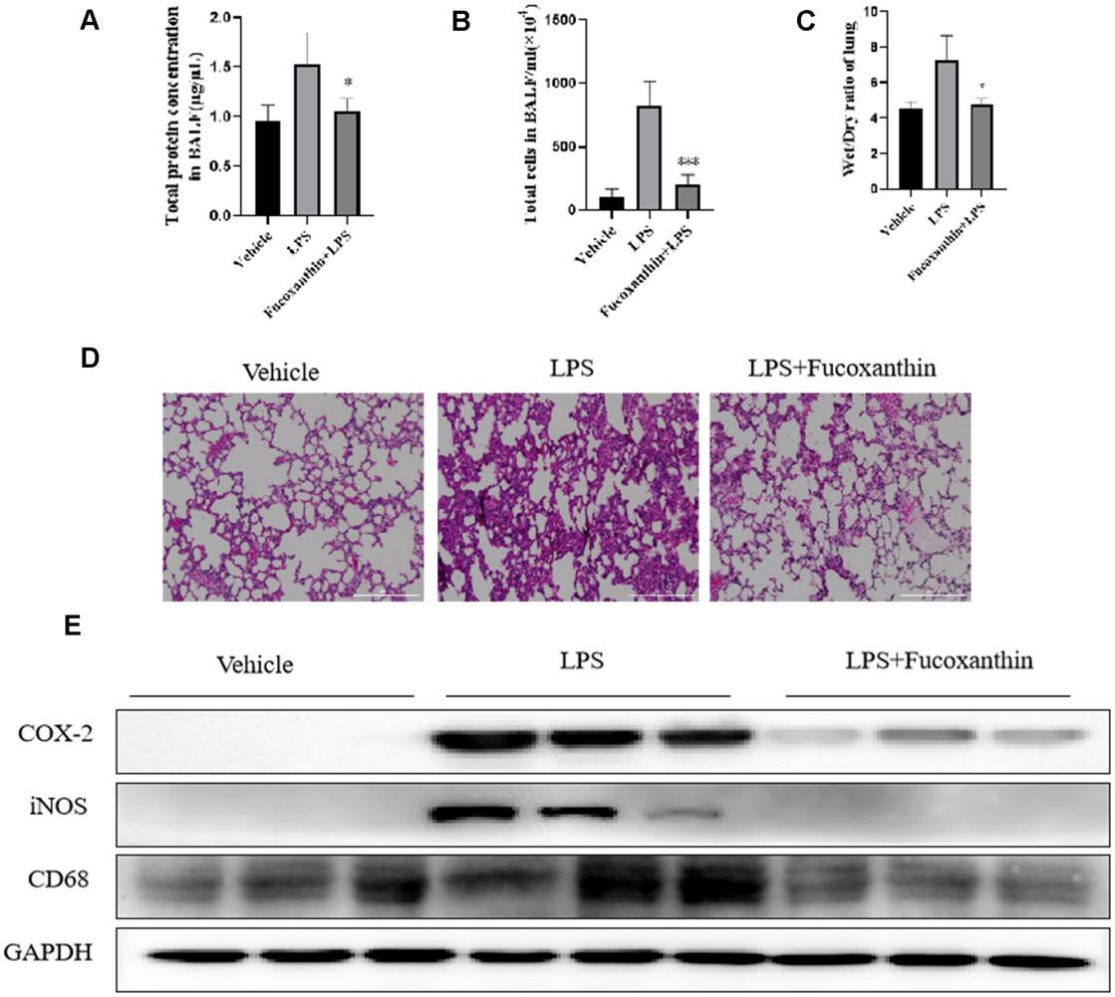
**Fucoxanthin attenuated the LPS-induced ALI in mice.** (**A**) The protein concentration in BALF. (**B**) The number of cells in BALF. (**C**) Wet/dry ratio. (**D**) Fucoxanthin attenuated the LPS-induced histopathological change in lung tissue (H&E staining). (**E**) Immunoblotting for COX-2, iNOS, and CD68 in the mice lung tissues. Data were presented as mean±SD. *P < 0.05, ***P < 0.01 *v. s* LPS group.

To further assess the anti-inflammatory effect of Fucoxanthin, the ALI inflammation mouse model was performed. As shown in Figure 7A, the protein concentration of BALF was significantly increased after LPS stimulation, whereas the pre-treatment of Fucoxanthin could reverse this increase. The LPS stimulation led to a significant increase in the number of cells in the BALF (Figure 7B), which was decreased by pre-treatment with Fucoxanthin. The lung wet/dry weight ratio was significantly increased after LPS-stimulation, compared with the vehicle. Nevertheless, pre-treatment with Fucoxanthin effectively decreased the lung wet/dry ratio (Figure 7C). We also found that LPS treatment dramatically increased the alveolar wall thickness, hemorrhage, alveolar collapse, and inflammatory infiltration in the lungs compared with the typical structure of mice lung tissues. As a comparison, the group pretreated with Fucoxanthin displayed very little histopathological changes (Figure 7D). We also detected the inhibitory effect of Fucoxanthin on macrophage lung infiltration marker CD68. Pre-treatment with Fucoxanthin significantly attenuated LPS-induced lung macrophage infiltration into the lung. Consistent with the results in vitro, Fucoxanthin significantly attenuated LPS-induced lung inflammation, as evidenced by COX-2 and iNOS expression in lung tissue (Figure 7E). These data indicate that Fucoxanthin attenuates the inflammation in LPS-treated ALI mouse model.

## DISCUSSION

In the present study, Fucoxanthin efficiently decreased reduced the expression of pro-inflammatory factors (COX-2, iNOS, IL-10, IL-6, TNF-α, and IL-1β), Fucoxanthin was further demonstrated to not only be able to inhibit LPS-stimulated COX-2 and iNOS protein expression, and it also is shown to have inhibitory effects on LPS-induced activation of NF-κB signaling. More importantly, Fucoxanthin effectively attenuates LPS-induced ALI in mice through lung tissue-specific anti-inflammatory effects. Interestingly, Fucoxanthin has been further shown to inhibit the TLR4/MyD88 signal pathway. Molecular docking experiments further confirmed that Fucoxanthin binds to the hydrophobic pocket of TLR4, which partially overlaps with the LPS binding site on TLR4. Taken together, these results suggest that Fucoxanthin may be a new candidate compound for the treatment of ALI.

ALI, characterized by increased excessive pulmonary inflammation [31]. Inflammation plays an essential role in the pathogenesis of ALI [32]. LPS is the main cell wall component of Gram-negative bacteria and is often used to induce the inflammatory model of ALI [33]. As the primary immune cells, macrophages are involved in the regulation of a variety of chronic inflammatory diseases, including ALI, by secreting a series of pro-inflammatory cytokines [34]. In this study, LPS-induced RAW264.7 cells were selected as an inflammatory model to evaluate the potential protective effects of drugs *in vitro* [35]. Previous studies have shown that LPS induces macrophages to release a large number of pro-inflammatory cytokines [34]. Our results show that Fucoxanthin can significantly inhibit LPS-induced inflammatory mediators.

NF-kB is one of the main factors of pro-inflammatory cytokines related to LPS-induced signal pathway [36]. As an upstream signal molecule of NF-κB, TLR4 plays an important role [37]. LPS usually activates the TLR4/MyD88/NF-κB pathway, which leads to the release of inflammatory cytokines. Therefore, inhibition of TLR4 dimerization is a new strategy for the treatment of inflammatory diseases. Many previous studies have shown that inhibition of LPS-induced TLR4 expression is another strategy for discovering new anti-inflammatory drugs related to TLR4 [38–40]. Our results showed that Fucoxanthin was further showed to inhibit. Our results also showed that Fucoxanthin inhibits the TLR4/MyD88/NF-κB signaling pathway. We further used molecular docking to detect whether Fucoxanthin

can be directly targeted to TLR4, thus inhibiting the TLR4 signaling pathway. Also, Fucoxanthin binds to the hydrophobic pocket of TLR4 and partially overlaps with the LPS binding site on TLR4, which further indicates that Fucoxanthin can directly target TLR4.

**Figure 8:**
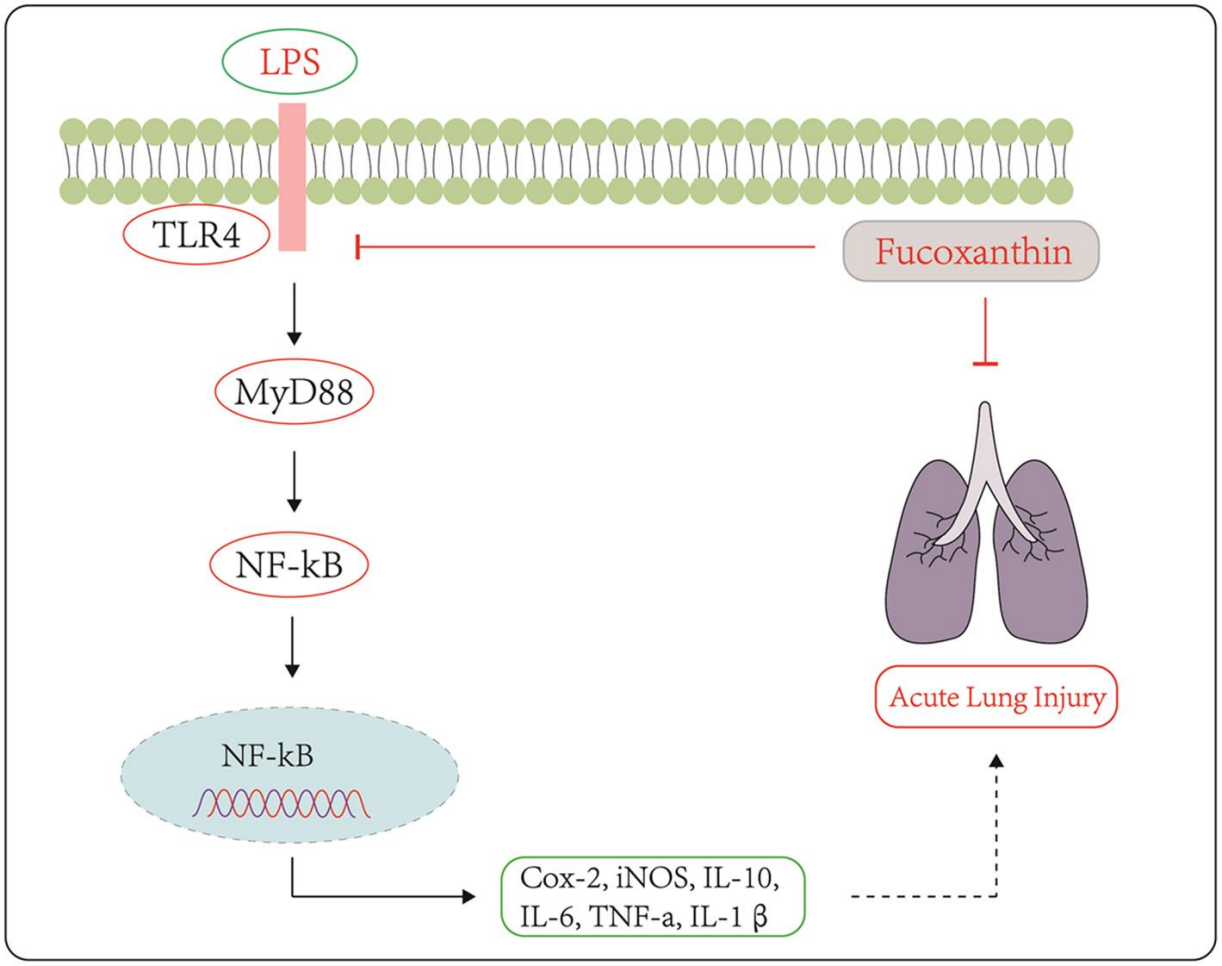
**Schematic diagram of the signaling pathways related to the anti-inflammatory effects of Fucoxanthin on LPS-induced ALI.**

To sum up, our study demonstrated that Fucoxanthin could protect LPS-induced ALI both *in vivo* and *in vitro*. The possible mechanism was illustrated in Figure 8. These results suggest that Fucoxanthin inhibits the TLR4/MyD88-mediated inflammatory signaling pathway by directly targeting TLR4, which suppresses LPS-induced inflammatory responses, and Fucoxanthin may be a candidate compound for the treatment of ALI.

## MATERIALS AND METHODS

### Materials

Fucoxanthin (purity > 99% cat#F6932) and Lipopolysaccharides (Escherichia coli O111: B4 cat#L2630) were purchased from Sigma-Aldrich (MO, USA).

### Cell culture

RAW264.7 cells were purchased from the American Type Culture Collection (ATCC). Cells were cultured in DMEM medium containing 10% FBS, 100 U/ml penicillin, and 100 μg/ml streptomycin. Cells were maintained at 37° C in a humidified atmosphere containing 95% air and 5% CO_2_.

### CCK-8 assay

RAW264.7 cells (1 × 10^4^ cells/plate) were seeded in 96-well plates and treated with Fucoxanthin at the indicated concentration for 24 h and then 10 μl CCK-8 reagent was added to each well, and the plates were incubated at 37º C for another 4 h. Next, the optical density was measured at a wavelength of 450 nm with the Bio-Rad (CA, USA) microplate reader.

### Immunofluorescence

Cells were cultured on glass slides and treated with LPS or Fucoxanthin for 24h. Cells were fixed by 4% formaldehyde for 10 minutes and followed by 0.3% Triton X-100 permeabilization for another 10 minutes, then blocked with 5% Goat Serum for 1 h. Subsequently, the cells were labeled with the primary antibody at 4° C overnight, washed with PBS three times, and labeled by Alexa Fluor 488 anti-rabbit at 1:200 dilution (Invitrogen™ cat#A12379) for 1 h at RT. After washed with PBS three times, cells were stained with DAPI at 1:1000 (Cell Signaling Technology, 4083) for 10 min, washed three times with PBS, and then subjected to image acquisition by Cytation 5 High content screening system (BioTek, VT, USA).

### Immunoblotting

The cells or tissues were lysed with RIPA lysis and extraction buffer on the ice for 10 min. The lysates were spun at 14,000g for 10 min at 4° C. BCA assay was used to quantify protein concentration. Samples with 20 μg of total protein were separated by 10% SDS-PAGE gels and transferred to PVDF membranes. Membranes were blocked for 1 h in TBS-T containing 5% non-fat dry milk and incubated with antibodies at 4° C overnight. After washing, blots were incubated with secondary antibodies (HRP-conjugated) for 1 h. Target proteins were detected using the LI-COR system for enhanced chemiluminescence. The following antibodies were used: COX-2 (CST, cat#12282, 1:1000 dilution), iNOS (CST, cat#D6B6S, 1:1000 dilution), TLR4 (Santa Cruz, cat# sc-293072, 1:1000 dilution), MyD88 (Santa Cruz, cat# sc-74532, 1:1000 dilution), CD68 (CST, cat#76437,1:1000 dilution), p-NF-κB p65 (CST, cat#3033,1:1000 dilution), GAPDH (CST, cat#5174,1:1000 dilution), secondary anti-rabbit HPR conjugated antibody (CST, cat#7074, 1:3000 dilution) and secondary anti-mouse HPR conjugated antibody (CST, cat#5127, 1:3000 dilution)

### Acquisition of fucoxanthin anti-inflammation targets

The structure of Fucoxanthin was searched from PubChem and imported into PharmMapper for the potential target using the pharmacophore mapping approach. The prediction target with Z value > 0.8 was retained, and the gene symbols were obtained and corrected as human protein by the Retrieve/ID mapping tool of Uniprot. We used the GeneCards database to identify the ALI-related targets with the phrase “acute lung injury” as a keyword. The potential targets of Fucoxanthin were compared with the related targets involved in ALI, and the prediction targets related to ALI were determined.

### Construction of PPI network and topological analysis in fucoxanthin against inflammation

In this study, we use the clustering analysis algorithm MCODE plug-in in Cytoscape to cluster the core-target PPI network, then used the Database for Annotation Visualization and Integration Discovery (David, https://david.ncifcrf.gov/) for KEGG enrichment analysis, and the screening criterion was P ≤ 0.05. Then we use Cytoscape to visualize the results.

### Molecular docking

The crystal structure of the ligand-free structure of the mouse TLR4/MD-2 complex was obtained from Protein Data Bank (PDB code: 5ijb), and the 3D structure of Fucoxanthin was obtained from PubChem (Compound CID: 5281239). Then, the molecular docking was based on the standard docking procedure for a flexible/rigid ligand with AutoDock 4. The result of docking is visualized by pymol software.

### Quantitative real-time PCR (qRT-PCR)

Total RNA was extracted using a Trizol assay kit. Briefly, RNA (1 μg) was subjected to qRT-PCR using a qPCR master mix kit. PCR amplification was performed by incorporating SYBR green (Roche). The primers for mouse iNOS, COX-2, TNF-α, IL-6, IL-10, IL-1β, and GAPDH were synthesized by Sangon Biotech (Shanghai). The following primer sequences were used: iNOS-F: GAGACAGGGAAGTCTGAAGCAC, iNOS-R: CCAGCAGTAGTTGCTCCTCTTC, COX-2-F: GCGACATACTCAAGCAGGAGCA, COX-2-R: AGTGGTAACCGCTCAGGTGTTG, TNF-α-F: GGTGCCTATGTCTCAGCCTCTT, TNF-α-R: GCCATAGAACTGATGAGAGGGAG, IL-6-F: TACCACTTCACAAGTCGGAGGC, IL-6-R: CTGCAAGTGCATCATCGTTGTTC, IL-1β-F: TGGACCTTCCAGGATGAGGACA, IL-1β-R: GTTCATCTCGGAGCCTGTAGTG, GAPDH-F: CATCACTGCCACCCAGAAGACTG, and GAPDH-R: ATGCCAGTGAGCTTCCCGTTCAG.

### ALI mouse model

Fucoxanthin was dissolved in 2% DMSO, 30%PEG-400, 2% Tween 80, and 66% PBS. Mice were randomized to the following three groups of seven mice: vehicle group, LPS group, Fucoxanthin + LPS group. Fucoxanthin + LPS group mice were pretreated with Fucoxanthin (10mg/kg) by i.v injection for 30 min before 5 mg/kg of LPS was administered by intratracheal instillation. The animals in the vehicle group received an equal volume of vehicles. After 6 h, mice were euthanized to collect the BALF, serum, and lung tissue samples. The BALF was collected by endotracheal intubation with normal saline for 3 times, and the maximum total amount of drip was 1ml.

### Lung wet/dry weight (W/D) ratio

Parts of the lungs were recorded to obtain the wet weight. Subsequently, the lungs were placed in an incubator at 60° C for 72 h to obtain the dry weight. The ratio of wet weight and dry weight (W/D) was calculated.

### Determination total protein concentration in Bronchoalveolar lavage fluid (BALF)

BALF was separated by centrifugal separation at 4° C and 3,000 rpm for 10 minutes. The total protein concentration of BALF was measured by a BCA assay kit.

### Histopathology

The lung tissue was fixed with 10% formalin and embedded in paraffin to make a 5 μm thick section. These sections are dewaxed with xylene and dehydrated in a series of alcohol solutions. The slides were stained with hematoxylin for 5min, then soaked in 1% acid ethanol for the 30s, and then rinsed with distilled water. The sections were stained with eosin for 3min and dehydrated in a graded series of alcohol. The mounted slides were then examined and photographed using Cytation 5 High content screening system (BioTek).

### Statistical analysis

The results were expressed as average ± SD. The statistical significance of the difference was evaluated by Student t-test or one-way ANOVA. The results shown represent at least three separate experiments. P < 0.05 was considered to be statistically significant between the two groups.
